# Clinical comparison between a percutaneous hydraulic pressure delivery system and balloon tamp system using high-viscosity cement for the treatment of osteoporotic vertebral compression fractures

**DOI:** 10.6061/clinics/2019/e741

**Published:** 2019-05-22

**Authors:** Jie Qin, Jianjun Li, Ying Liu, Bo Zhao, Hui Dong, Bo Dong, Rui Zhang, Ning Ning, Xin Zhang, Feng Cui, Haopeng Li, Xijing He, Dong Wang

**Affiliations:** I The Department of Orthopedics, the Second Affiliated Hospital of Xi'an Jiaotong University, Xi'an, Shaanxi Province, P. R. of China; II The Key Laboratory of Biomedical Information Engineering of the Ministry of Education, School of Life Science and Technology, Xi'an Jiaotong University, Xi'an, Shaanxi Province, P. R. of China; III The Department of Education, the Second Affiliated Hospital of Xi'an Jiaotong University, Xi'an, Shaanxi Province, P. R. of China; IV The Department of Orthopedics, the Affiliated Hospital of Shaanxi University of Traditional Chinese Medicine, Xi'an, Shaanxi Province, P. R. of China; V The Department of Orthopedics, Tangdu Hospital, Xi'an, Shaanxi Province, P. R. of China; VI Xi'an Honghui Hospital, Xi'an Jiaotong University, Xi'an, Shaanxi Province, P. R. of China; VII Foreign Language School, Northwest University, Xi'an, Shaanxi Province, P. R. of China

**Keywords:** Osteoporotic Vertebral Compression Fractures, Percutaneous Kyphoplasty, Percutaneous Vertebroplasty, Hydraulic Pressure Delivery System, Balloon Tamp System, High-Viscosity Cement

## Abstract

**OBJECTIVES:**

Osteoporotic vertebral compression fractures (OVCFs) affect the elderly population, especially postmenopausal women. Percutaneous kyphoplasty is designed to treat painful vertebral compression fractures for which conservative therapy has been unsuccessful. High-viscosity cement can be injected by either a hydraulic pressure delivery system (HPDS) or a balloon tamp system (BTS). Therefore, the purpose of this study was to compare the safety and clinical outcomes of these two systems.

**METHODS:**

A random, multicenter, prospective study was performed. Clinical and radiological assessments were carried out, including assessments of general surgery information, visual analog scale, quality of life, cement leakage, and height and angle restoration.

**RESULTS:**

Using either the HPDS or BTS to inject high-viscosity cement effectively relieved pain and improved the patients' quality of life immediately, and these effects lasted at least two years. The HPDS using high-viscosity cement reduced cost, surgery time, and radiation exposure and showed similar clinical results to those of the BTS. In addition, the leakage rate and the incidence of adjacent vertebral fractures after the HPDS treatment were reduced compared with those after treatment using the classic vertebroplasty devices. However, the BTS had better height and angle restoration abilities.

**CONCLUSIONS:**

The percutaneous HPDS with high-viscosity cement has similar clinical outcomes to those of traditional procedures in the treatment of vertebral fractures in the elderly. The HPDS with high-viscosity cement is better than the BTS in the treatment of mild and moderate OVCFs and could be an alternative method for the treatment of severe OVCFs.

## INTRODUCTION

Osteoporotic vertebral compression fractures (OVCFs) are a common cause of pain and disability in people over 50 years old, especially in elderly women. Osteoporosis affects 44 million Americans 1 and 27.5 million Europeans 2. In the year 2000, the number of osteoporotic fractures worldwide was estimated to be nine million, of which 1.4 million were vertebral compression fractures 3. Percutaneous kyphoplasty (PKP) and percutaneous vertebroplasty (PVP) are currently used for the management of painful OVCFs that fail to respond to conventional therapies.

Many studies have compared the clinical results of PVP and PKP. Both of these procedures are based on the injection of medical cement into a collapsed vertebral body, resulting in immediate and sustained pain relief 4-6. The main complication and cause for concern among surgeons and patients is the cement leakage. Leakage, which is the extravasation of cement beyond the confines of the collapsed vertebral body, results in not only minor complications without special therapy and short- or long- consequences but also major complications such as urgent laminectomy decompression and embolism.

Classical PVP consists of the injection of a low-viscosity cement into the fractured vertebra using a push rod at a high pressure, which results in either a small amount of injected cement or more cement extravasation. To improve this situation, PKP is performed in which an inflatable device such as a balloon is inserted. Using this method, even a ball of high-viscosity cement could be injected into the fractured vertebral body and inflated to elevate both endplates to create a cavity. In addition, the cement is injected into the cavity at a low pressure, significantly decreasing the potential of extravasation 7. Hulme et al. reviewed the clinical studies regarding these two procedures and found that the mean rate of cement leakage due to PVP was of 41% and that due to PKP was 9% 8. Other evidence confirmed that PKP results in less cement leakage than does PVP 9,10. A meta-analysis reported by Eck JC et al. showed greater improvement in pain scores when PVP was used instead of PKP, but there was less new fracture after PKP (14.1%) than after PVP (17.9%) and less cement leak risk after PKP (7.0%) than after PVP (19.7%) 11.

Some studies reported that an increase in viscosity from low to medium can reduce cement extravasation 12-14, which is completely avoided when poly (methyl methacrylate) (PMMA) reaches high viscosity and achieves a dough-like consistency. Nevertheless, the classical syringe, cannula, and push rod cannot inject a cement with such high viscosity. To solve this problem, a new hydraulic cement delivery system was developed that can inject highly viscous cement into the collapsed vertebral body without using balloon expansion to create a cavity. By using this system, the cement extravasation rate could probably decrease, reducing complications arising from the cement leakage. The balloon tamp system (BTS), which holds the cement in the vertebral body, was considered the critical reason for the difference in leakage rate 15. Moreover, cement viscosity and injection volume were considered other main factors influencing cement extravasation 16. Therefore, we wondered if we could inject high-viscosity cement using a hydraulic pressure delivery system (HPDS) without the need to create a cavity with the BTS. To answer this question and to compare the safety and efficacy of HPDS and BTS using the high-viscosity cement in the treatment of OVCF, we performed the following prospective clinical study.

## MATERIALS AND METHODS

### Study design and participants

**Figure 1:**
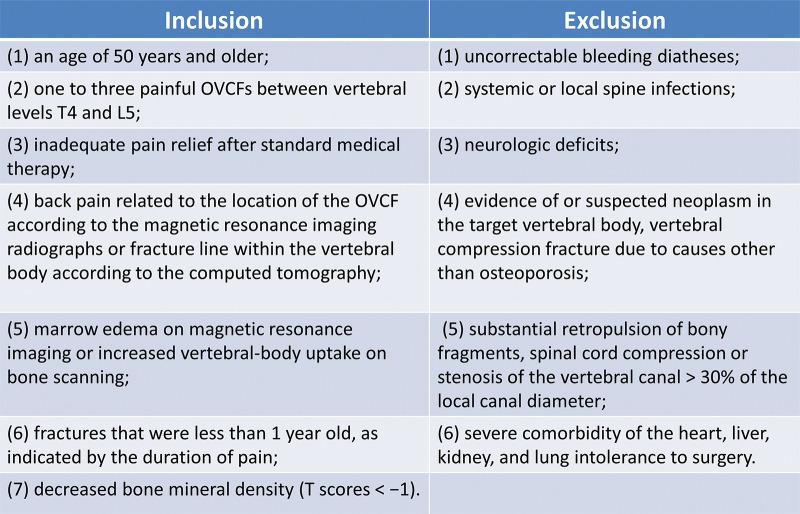
Inclusion and exclusion criteria.

A randomized, controlled, four-center prospective study was performed to compare the clinical outcomes of HPDS or BTS using high-viscosity cements for relieving pain and restoring the physical functions in people with painful OVCFs. The Institutional Review Board approved this study, and the patients provided informed consent prior to the study. Four hospitals were involved in this study, and enrollment started in April 2014 and continued through January 2016; the follow-up period was two years. As described previously 17,18, the study inclusion and exclusion criteria are shown in Figure 1.

### Interventions and surgical procedures

**Figure 2:**
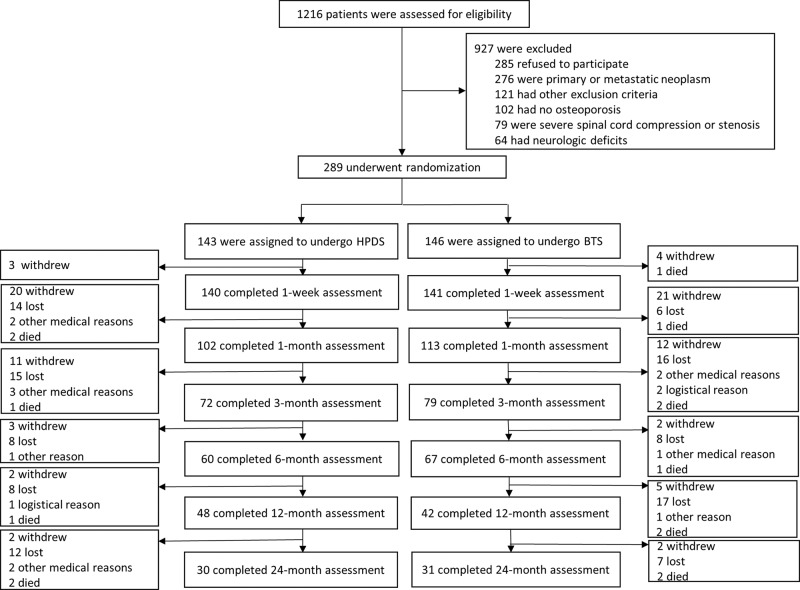
Participant enrollment, assigned Intervention, and follow-up.

At baseline, the demographic and clinical information, including age, gender, smoking status, bone mineral density, medicine use, fracture history and vertebral fracture nature, was collected 1 day to 1 week before randomization. All surgeons in this trial belonging to the four participating hospitals were experienced and had performed more than 50 procedures. The participants were randomly assigned to undergo either HPDS or BTS surgery, and steps were taken to make sure that the participants remained unaware of their assigned intervention (Figure 2).

**Figure 3:**
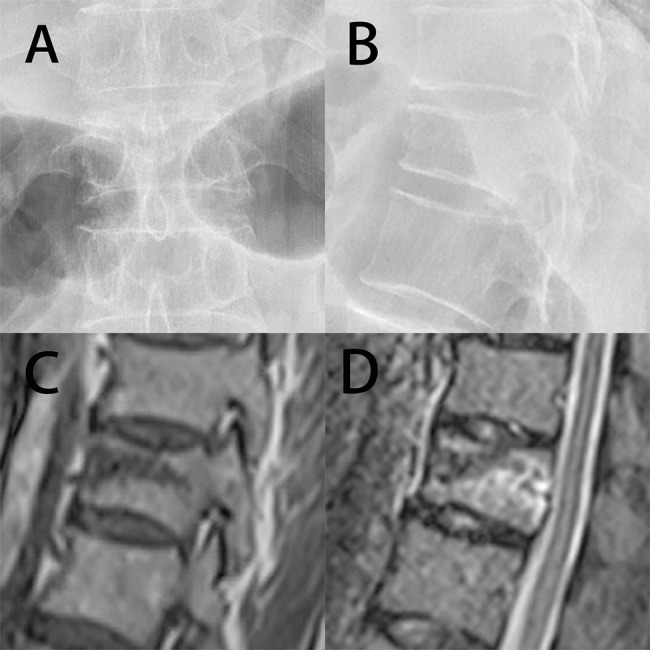
Typical X-ray and magnetic resonance (MR) images of OVCFs. A and B, Posterior-anterior and lateral X-ray films showing a compression fracture in T12. Both endplates were fractured. C and D, MR images showing marrow edema in the fractured vertebral body.

The prone position was used when sedation and analgesia were induced in the patients. After the fractured vertebral body and its corresponding pedicles were located using fluoroscopy (Figure 3), the surgical procedures were conducted under local or general anesthesia. All participants received cephalothin administered intravenously 30 min before puncture. Under fluoroscopic guidance, beveled injection needles were punctured into the thoracic vertebra or the vertebral arch in the lumbar vertebra. Then, the patients underwent the HPDS or BTS procedure according to their random assignment.

**Figure 4:**
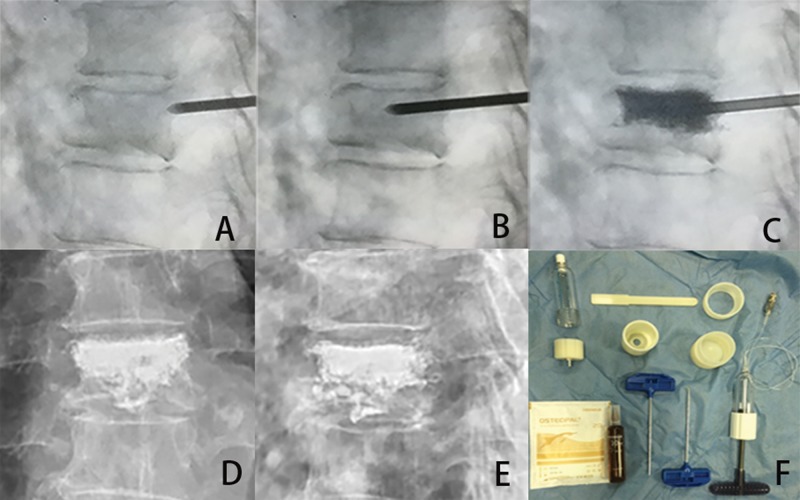
HPDS surgical procedure for the treatment of a 79-year-old female patient with T8 vertebral body fracture. A, A puncture needle enters into the fractured T8 vertebra via the left pedicle. B, Lateral radiograph showing the puncture needle tip in the anterior column of the vertebral body. C, High-viscosity bone cement is injected into the fractured vertebral body. The lateral radiograph shows the filling of the vertebral body with high-viscosity cement. D and E, Lateral and posterior-anterior X-ray films after the HPDS surgical procedure. F, The HPDS device and high-viscosity cement.

For the HPDS procedure, once the needle was punctured into the center of the vertebra, a high-viscosity bone cement was prepared by mixing the powder and liquid components until a paste was formed. Then, PMMA was loaded into a bone cement perfusion apparatus (Zhongshan Shiyitang Medical Devices Co., Ltd., China) and manually injected into the target vertebra using the HPDS under constant lateral fluoroscopic guidance (Figure 4).

**Figure 5:**
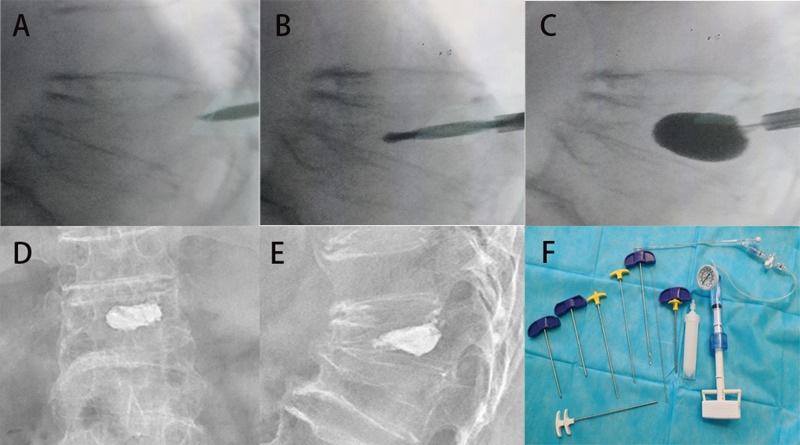
BTS surgical procedure for the treatment of a 75-year-old male patient with T12 vertebral body fracture. A, A puncture needle enters into the wedge T12 vertebra via the left pedicle. B, A bone drill was inserted in the fractured vertebral body to drill a circular hole as a working channel. C, An expandable cannula was inserted into the fractured vertebral body, and the balloon was slowly inflated. D and E, Posterior-anterior and lateral X-ray films after the BTS surgical procedure. F, The BTS instruments.

For the BTS procedure, a bone drill was used to create a working channel according to well-established methods. Next, an inflatable balloon tamp (Shanghai Kinetic Medical Co., Ltd., China) was inserted into the center of the vertebral body, followed by sequential pressure-controlled inflation of the balloon to create a cavity. The bone cement was then mixed and manually injected into the cavity (Figure 5) 19.

In both groups of patients, the cement injection was stopped when 1) the cement reached the posterior wall of the fractured vertebral body, 2) a substantial resistance was detected during injection, 3) the presence of radiologically adequate filling was reached, or 4) a potential leakage was suspected.

Following the surgery, the patients rested for 6 hours, and their vital signs and neurologic symptoms were monitored for up to 24 hours.

### Clinical and radiological assessments

The patients received written evaluation questionnaires that were mailed, emailed, or shared via WeChat 3d before and 1 w, 3 m, 6 m, 12 m and 24 m after the surgery.

The clinical assessment included: (1) surgery information including the average surgery time (incision to suturing/number of treated vertebral levels), X-ray exposure times during the surgery, injected cement dosage, and cost of hospital stay; (2) a visual analog scale (VAS) for overall pain; (3) the modified Roland-Morris Disability Questionnaire (RDQ) 17,20,21; (4) the European Quality of Life–5 Dimensions (EQ–5D) scale^17^,^22^; (5) the Short Form-36 General Health Survey (SF-36) including the physical component summary (PCS) and the mental component summary (MCS) for the quality-of-life evaluation; (6) the Oswestry Disability Index (ODI) for functional assessment; and (7) surgery related complications. The primary outcome measures were the VAS scores for back pain and the modified RDQ.

The radiological assessment included: (1) cement leakage; (2) vertebral height restoration; (3) vertebral wedge angle; and (4) adjacent vertebral fracture at final follow-up. The vertebral height restoration percentage was calculated as follows 23: vertebral height restoration percentage = (anterior or middle height postoperation – anterior or middle height preoperation) / (anterior or middle height preoperation) × 100%. The vertebral wedge angle was equal to the angle between the upper and lower endplates of the vertebral body before and after the operation 24.

### Statistical analysis

The continuous variables were analyzed using Student's *t*-test. The categoric variables were assessed using the χ^2^ test. The results are presented as the mean±SD. A *p* value <0.05 was considered significant. SPSS 22.0 was used for all statistical analyses.

## RESULTS

### Patient information

A total of 1216 potential patients were identified from 2014 to 2016, but only 289 of those met the inclusion criteria and underwent randomization (143 in the HPDS group and 146 in the BTS group). Three participants (2%) in the HPDS group and five (3%) in the BTS group were lost to the 1 w follow-up. At the 2-year follow-up, complete data were available for 30 of the 143 participants (21%) in the HPDS group and 31 of the 146 participants (21%) in the BTS group. The baseline characteristics of the two groups were similar and are summarized in Table 1. There was no statistically significant difference in the bone mineral density T-score, medicine usage, severity of fracture, fracture distribution and demographic data such as gender and age. No significant difference was found between the two groups regarding the VAS and quality-of-life scores, such as RDQ, EQ-5D, SF-36 and ODI before surgery. If the participants were lost to follow-up at one time point, they were not recorded in the following follow-up.

### General surgery parameters

The mean surgery times were 35 min for the HPDS group and 45 min for the BTS group (*p*<0.05). The mean X-ray exposure times were 45 times for the HPDS group and 72 times for the BTS group, and the difference was significantly different (*p*<0.05). The surgery time and X-ray exposure time of the puncture procedures were longer than those of the other procedures, and no significant difference was observed between the HPDS and BTS groups. The surgery time and X-ray exposure time of the balloon augmentation procedure were longer for the BTS group than for the HPDS group. The injected cement volumes were 3.5±1.1 mL for the HPDS group and 4.7±1.5 mL for the PKP group. Thus, the injected cement dose used to perform BTS was more than the dose injected using the perform HPDS. However, a larger dose of cement than that injected when performing the classical PVP with low-viscosity cement was used to inject high-viscosity cement using the HPDS. The BTS was more expensive than the HPDS because of the high cost of the balloon tamp (Table 2).

### Pain and functional evaluations

**Figure 6:**
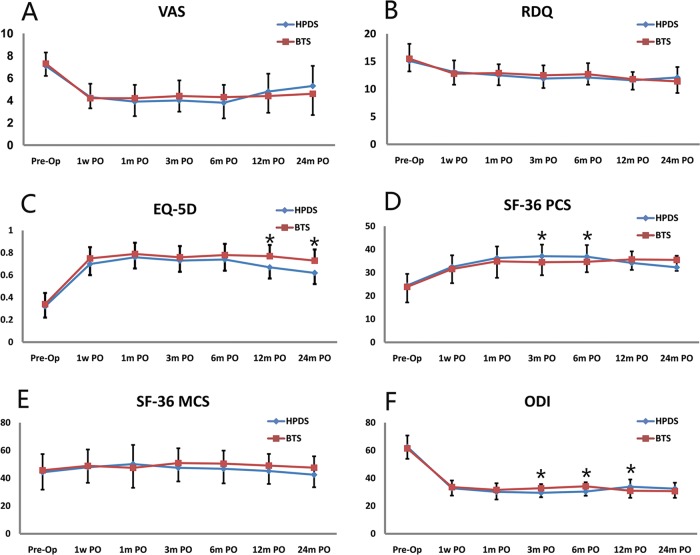
Scores of VAS (A), RDQ (B), EQ-5D(C), SF-36 PCS (D), SF-36 MCS (E) and ODI (F) with respect to time and intervention group (*p<0.05).

The pain and functional evaluations were recorded and are shown in Table 3. VAS and RDQ were the primary outcomes. No significant difference between the HPDS and BTS groups was found regarding the primary outcome of the overall pain in VAS and RDQ postoperation (PO). A significant difference (*p*<0.05) in the VAS and RDQ scores was observed before and 1 w after surgery, indicating that all patients experienced pain relief and mobility improvement immediately after surgery. Moreover, the 2-year follow-up results showed that the VAS scores PO did not change. Therefore, both HPDS and BTS using high-viscosity cement resulted in significant pain relief and the improved recovery of functional abilities. The mean reductions in the VAS scores of the HPDS and BTS groups were 2.9±2.1 and 3.3±2.3, respectively, but the difference between these values was not statistically significant. No significant difference in any other secondary parameter was observed between these two groups over time, except for SF-36 PCS 3 m and 6 m PO, EQ-5D 12 m and 24 m PO, and ODI 3 m, 6 m and 12 m PO. The use of analgesics, NSAIDS or opioids decreased over the 12 months follow-up, with no significant difference between the two groups. However, analgesic use increased after 24 months compared with that after 12 months, and the VAS increased after 24 months compared with that after 12 months. The reason for these differences might be the new fracture formation. The RDQ scores before surgery were 15.1±3.1 for the HPDS group and 15.5±2.3 for the PKP group, and these values decreased to 13.1±2.1 and 12.8±2.0 one week after surgery, respectively. Both the HPDS and BTS groups showed sustained improvement for at least 2 years, and similar improvement was observed in these two groups. There was no significant difference in the secondary outcomes such as EQ-5D, SF-36 and ODI (Figure 6).

### Radiological evaluations

The X-ray films were analyzed to measure the cement extravasation, height restoration percentage, wedge angles and adjacent vertebral fractures after surgery (Table 4). The leakage rate in the BTS group was lower than that in the HPDS group. Better height restoration in the anterior and middle vertebral body and wedge angle was observed after the BTS intervention. Therefore, when high-viscosity cement was used, both the HPDS and BTS restored the height of the vertebral body significantly. The BTS group had significantly higher height and wedge angle restoration than the HPDS group (*p*<0.05). Therefore, both the HPDS and BTS treatments enabled height restoration, although the BTS treatment resulted in a better outcome. The vertebral anterior and middle height restoration percentages for severe fractures in the HPDS group were 5.6±4.4 and 10.3±4.9, respectively, while those in the BTS group were 15.6±6.1 and 27.5±12.1, respectively, suggesting that the BTS resulted in better height restoration in severe fractures than in mild and moderate fractures. The percentages for adjacent vertebral fractures were 13% after the HPDS treatment and 16% after the BTS treatment.

## DISCUSSION

OVCFs are usually painful for patients and affect more women, especially postmenopausal women, than men. PVP and PKP are currently widely used vertebral augmentation procedures for treating painful OVCFs when conservative therapy is not effective. Vertebral body augmentation stabilizes the fractured vertebral body through infused PMMA without increasing mortality 4,25. PMMA monomers used to reduce pain are cardiotoxic, while the polymerization of PMMA monomers causes thermal injuries to the surrounding tissues. High-viscosity cement injections lower the potential for cardiovascular complications 26 and have been shown to significantly reduce the leakage rate and frequency of leakage-related complications compared with low-viscosity cement injections 27-29. Thus, the use of high-viscosity cement in a new device for vertebral augmentation could be promising.

The surgery time for the HPDS group was significantly shorter than that of the BTS group. The time spent on the puncture and injection steps was almost the same for these two groups. The time used to apply the balloon was longer for the BTS group than for the HPDS group. In addition, the balloon application and expansion step for the BTS resulted in longer X-ray exposure time; injecting high-viscosity cement using the new HPDS reduced the need for bone tamps in the BTS, significantly reducing the number of steps in the procedure and the surgery time.

Both the HPDS and BTS resulted in pain reduction immediately after the operation that lasted at least 2 years. The VAS in both groups was better than that before the surgery, and no significant differences were found between these two groups in 1 w, 1 m, 3 m, 6 m and 12 m PO. Furthermore, after reviewing the PO data in the mild and moderate fracture subgroups, we found no significant difference between the HPDS and BTS; however, in the severe fracture subgroup, the VAS in the BTS (4.2±0.9 and 5.3±1.4) was significantly better than that in the HPDS (4.9±0.8 and 5.9±1.3) in 12 m and 24 m PO, respectively. These findings indicated that the BTS may result in better pain relief in patients with severe fracture 1 year PO than the HPDS. We used the RDQ, EQ-5D, SF-36 and ODI to evaluate life quality, disability and functional recovery. A clear improvement in the life quality of both HPDS and BTS groups was observed that lasted at least 2 years. There was no significant difference between the HPDS and BTS groups in the PO scores of RDQ, SF-36 MCS, ODI, most of the SF-36 PCS and EQ-5D.

The HPDS using high-viscosity cement significantly decreased the leakage rate compared to PVP using low- to medium-viscosity cement^29^. PKP had excellent abilities in vertebral height and kyphotic angle restoration. The vertebral height and kyphotic angle restoration in the HPDS group were less than those in the BTS group because of the position of the patient, although the cement injected into the vertebral body slightly restored the height and angle. The inflation of a balloon tamp inside the vertebral body plays an important role in height and angle restoration. This observation explains why the BTS showed better height and angle restoration abilities, especially in patients with severe fracture. These abilities might result in better VAS scores in severe fracture patients treated by the BTS, even though there was no significant difference between the HPDS and BTS groups in the quality-of-life scores.

The cemented vertebral bodies and the increased height of the collapsed vertebra changed the biomechanical property of and increased the load on the adjacent vertebral fracture. Literature reports on the incidence of sustained adjacent fracture after PVP or PKP show conflicting findings. The risks for new adjacent fractures are lower after PVP than after PKP 30,31, while some systematic reviews showed that PVP resulted in higher adjacent factures than did PKP 11,32; finally, some other reviews reported that the fracture risks of PVP and PKP were similar 33,34. The adjacent fracture may be a consequence of osteoporosis. Komemushi A et al. 35 thought that cement extravasation into the intervertebral space increases the incidence of adjacent fractures, which might explain why there was less adjacent vertebral fracture in the HPDS group than in the BTS group in this study. Since the classic PVP procedure usually induces much higher leakage than the BTS, the HPDS using high-viscosity cement decreased the leakage rate, thus decreasing the incidence of adjacent fractures. This high-viscosity cement injection system is similar to the Confidence Spinal Cement System used by Georgy, B.A. In a?single-center retrospective review of the Confidence device using high-viscosity cement 36, a similar leakage rate was found in the Confidence device and PKP groups. Herein, we found that the leakage rate in the HPDS group was higher than that in the BTS group but was significantly lower than that reported for PVP using low- to medium-viscosity cement 29,36. This evidence demonstrates that the HPDS with high-viscosity cement effectively reduces the leakage rate compared with the classic PVP device using low- to medium-viscosity cement.

It was difficult to determine whether surgery using the HPDS was better than that using the BTS because better designed random control trials are needed. In contrast with other studies 29,36 related to PVP, this is a random, prospective study. Moreover, some limitations exist in this study. First, some participants were lost to the long-term follow-up over time. Second, large sample numbers are need for further investigation.

## CONCLUSION

Despite the difficulty of evaluating which one of the two surgeries considered in this study is better, the overall evidence shows that the use of the HPDS with high-viscosity cement has similar outcomes to the BTS in the clinical treatment of OVCFs. We suggest using the HPDS to treat mild and moderate OVCFs and choosing the HPDS as an alternative method to treat severe OVCFs.

## AUTHOR CONTRIBUTIONS

Qin J performed the study, collected and analyzed the data, and wrote the manuscript. Li J performed the study and wrote the manuscript. Liu Y collected the data. Zhao B, Dong B, Zhang R, Ning N, Cui F, Li H participated in the study. Dong H analyzed the data and elaborated the tables. Zhang X participated in the manuscript writing. He X conceived and designed this study and revised the manuscript. Wang D conceived and designed the study and revised the manuscript.

## Figures and Tables

**Table 1:** Baseline characteristics and clinical data of the patients.

Characteristic	HPDS	BTS
Age (mean±SD)	72.1±9.2	71.1±8.6
Female - no. (%)	104 (73)	110 (75)
Current smoker - no. (%)	23 (16)	21 (14)
Bone mineral density T-score	-2.66±0.89	-2.72±0.76
Medication for osteoporosis - no. (%)		
Any	128 (90)	135 (92)
Calcium supplements	117 (82)	115 (79)
Vitamin D	74 (52)	70 (48)
Bisphosphonates	36 (25)	32 (22)
Previous vertebral fractures - no. (%)	53 (37)	50 (34)
Pain duration (week, mean±SD)	6.8±3.2	7.1±4.1
VAS during the past 24 hours (mean±SD)	7.1±1.2	7.3±1.1
Use of analgesic (NSAIDS or opioids) - no. (%)	105 (73)	112 (77)
Severity of fracture - (%)		
Mild	44 (31)	42 (29)
Moderate	69 (48)	76 (52)
Severe	30 (21)	28 (19)
Number of treated vertebral levels - no. (%)	201 (100)	218 (100)
1	98 (69)	95 (65)
2	32 (22)	30 (21)
3	13 (9)	21 (14)
Fracture distribution - no. (%)		
Thoracic	145 (72)	168 (77)
Lumbar	56 (28)	50 (23)
RDQ (mean±SD)	15.1±3.1	15.5±2.3
EQ-5D (mean±SD)	0.32±0.29	0.34±0.31
SF-36 (mean±SD)		
PCS	24.5±7.2	23.9±6.7
MCS	44.3±13.1	45.7±13.9
ODI (mean±SD)	62.6±8.2	61.5±7.5

No significant differences in the baseline data between the two groups were found.

**Table 2:** Surgery parameters (mean±SD, minimum-maximum).

	HPDS	BTS
Average surgery time per vertebra (min)	35±19 (15-52)	45±21* (25-71)
X-ray exposure times	45±22 (31-122)	72±25* (65-182)
Injected cement dosage per vertebra (ml)	3.5±1.1 (1.3-5.2)	4.7±1.5* (2.4-7.3)
Cost (dollars)	4570±860 (3610-5680)	5230±980* (4250-7820)

*p<0.05 *versus* HPDS.

**Table 3:** Clinical assessments (mean±SD).

	Parameters	HPDS	BTS
Preoperation	VAS	7.1±1.2	7.3±1.1
	RDQ	15.1±3.1	15.5±2.3
	EQ-5D	0.32±0.29	0.34±0.31
	SF-36 PCS	24.5±7.2	23.9±6.7
	SF-36 MCS	44.3±13.1	45.7±13.9
	ODI	62.6±8.2	61.5±7.5
	Use of analgesic - No. (%)	105 (73)	112 (77)
1 week PO	VAS	4.3±1.2	4.2±0.9
	RDQ	13.1±2.1	12.8±2.0
	EQ-5D	0.70±0.31	0.75±0.34
	SF-36 PCS	32.5±5.6	31.6±6.1
	SF-36 MCS	47.9±12.8	48.9±12.2
	ODI	32.8±5.6	33.6±6.1
	Use of analgesic - No. (%)	57 (41)	61 (43)
1 month PO	VAS	3.9±1.5	4.2±1.6
	RDQ	12.5±2.0	12.9±2.2
	EQ-5D	0.76±0.29	0.79±0.35
	SF-36 PCS	36.3±6.5	34.9±7.1
	SF-36 MCS	50.2±13.8	47.5±14.4
	ODI	30.2±6.2	31.6±6.9
	Use of analgesic - No. (%)	33 (32)	35 (31)
3 months PO	VAS	4.0±1.8	4.4±1.4
	RDQ	11.9±2.4	12.5±2.3
	EQ-5D	0.73±0.31	0.76±0.32
	SF-36 PCS	37.1±5.2	34.5±5.6*
	SF-36 MCS	47.5±14.1	50.9±13.2
	ODI	29.5±6.3	32.8±6.5*
	Use of analgesic - No. (%)	17 (24)	16 (20)
6 months PO	VAS	3.8±1.6	4.3±1.9
	RDQ	12.1±2.6	12.7±1.9
	EQ-5D	0.74±0.35	0.78±0.33
	SF-36 PCS	36.9±4.9	34.9±4.5*
	SF-36 MCS	46.8±13.1	50.5±14.2
	ODI	30.4±6.7	34.2±6.8*
	Use of analgesic - No. (%)	9 (15)	11 (16)
12 months PO	VAS	4.8±1.6	4.4±1.5
	RDQ	11.6±1.5	11.8±1.9
	EQ-5D	0.67±0.23	0.77±0.21*
	SF-36 PCS	34.2±4.8	35.5±4.5
	SF-36 MCS	45.2±12.3	49.1±13.2
	ODI	33.9±5.2	31.0±5.1*
	Use of analgesic - No. (%)	6 (13)	4 (10)
24 months PO	VAS	5.3±1.8	4.6±1.9
	RDQ	12.1±1.9	11.4±2.1
	EQ-5D	0.62±0.19	0.73±0.22*
	SF-36 PCS	32.3±5.3	34.4±4.7
	SF-36 MCS	42.5±13.3	47.6±14.1
	ODI	32.5±4.3	30.7±4.8
	Use of analgesic - No. (%)	6 (20)	7 (23)

*p<0.05 *versus* HPDS.

**Table 4:** Radiological assessments.

	HPDS	BTS
Cement leakage - n(%)/(Total treated levels)	21 (10)	14 (6)
Vertebral height restoration (%)		
Anterior	6.6±4.2	10.6±8.1*
Middle	9.2±4.1	20.5±14.8*
Wedge angle (°)		
Preoperation	20.1±10.2	22.6±12.2*
1 week PO	14.4±8.1	11.4±6.6*
Final follow-up	14.5±8.6	11.1±7.1*
Adjacent vertebral fractures - n(%)/(Total treated levels)	26 (13)	35 (16)

*p<0.05 *versus* HPDS.
